# Association of circadian syndrome with nonalcoholic fatty liver disease and liver fibrosis: A cross-sectional study based on NHANES 2017 to 2020.03 data

**DOI:** 10.1097/MD.0000000000047950

**Published:** 2026-03-06

**Authors:** Wenzhao Guo, Sijia Li, Bingjiu Lu, Xinghai Yue, Yuhang Gao, Meng Mi, Wenting Zhao, Ran Zhao, Guanhua Lv

**Affiliations:** a Graduate School, Liaoning University of Traditional Chinese Medicine, Shenyang, China; b Department of Hepatology, The Affiliated Hospital of Liaoning University of Traditional Chinese Medicine, Shenyang, China; c Department of Intervention, Shenyang Sixth People’s Hospital, Shenyang, China; d Department of Chinese and Western Medicine Combined, Handan First Hospital, Handan, China; e Department of Gastroenterology, The Second Affiliated Hospital of Liaoning University of Traditional Chinese Medicine, Shenyang, China.

**Keywords:** CircS, liver fibrosis, NAFLD, NHANES

## Abstract

The circadian rhythm syndrome (CircS) is closely linked to metabolism; however, its association with nonalcoholic fatty liver disease (NAFLD) and liver fibrosis remains inadequately explored. This study aims to explore the association of CircS with NAFLD and liver fibrosis. A cross-sectional analysis was performed using data from the National Health and Nutrition Examination Survey, and 3 multivariable logistic regression models were constructed to examine the association of CircS with NAFLD and liver fibrosis. Additionally, subgroup and interaction analyses were conducted to identify potential influencing factors. The study enrolled a total of 2597 participants. In models adjusted for all covariates, the odds ratios (OR) of CircS for NAFLD and liver fibrosis were (OR = 4.85, 95% CI: 3.99–5.89) and (OR = 1.73, 95% CI: 1.29–2.34), respectively. Subgroup and interaction analyses revealed a stronger positive association of CircS with NAFLD and liver fibrosis in younger age groups (<60 years) (*P* = .049, *P* = .004). Furthermore, CircS participants with diabetes showed a stronger positive correlation with NAFLD (*P* = .002). This indicates that in U.S. adults, CircS is significantly associated with the prevalence of both NAFLD and liver fibrosis. The association between CircS and NAFLD is stronger in individuals under 60 years of age and those with diabetes, while the association of CircS with liver fibrosis is more pronounced in individuals under 60 years of age.

## 1. Introduction

Nonalcoholic fatty liver disease (NAFLD), affecting approximately 25% of the global population, is a persistent liver disorder strongly associated with metabolic dysfunctions.^[1,2]^ NAFLD can progress to liver fibrosis, and although liver biopsy is currently considered the most definitive method for diagnosing both conditions, its invasive nature makes it unsuitable for repeated use within a short timeframe, posing significant limitations in clinical practice.^[3,4]^ Liver ultrasound transient elastography (LUTE) offers a noninvasive technique to quantify liver steatosis and fibrosis, frequently serving as a standard tool for monitoring therapeutic outcomes and tracking disease progression.^[5]^

The circadian rhythm is a biological process regulated by light/dark cycles, typically following a 24-hour cycle in humans. It governs sleep, mood, feeding, metabolism, immunity, and nearly all other physiological processes, allowing the body to maintain rhythmic behaviors even in the absence of light.^[6]^ Circadian rhythm disruption occurs when the body’s internal biological clock is desynchronized, often caused by changes in light exposure or behavioral shifts such as shift work or jet lag.^[7]^ These disturbances can disrupt cellular functions at the molecular level, leading to metabolic dysregulation and the development of metabolic diseases.^[8,9]^ Research using animal models has demonstrated that disturbances in circadian rhythms contribute to the development of insulin resistance, impaired glucose tolerance, obesity, and NAFLD.^[10]^ Clinically, NAFLD is often associated with irregular sleep patterns, shift work, jet lag, and insufficient sleep.^[11,12]^

Circadian syndrome (CircS) is defined by the presence of at least 4 persistent health issues, such as lipid metabolism disorders, depression, abdominal obesity, hypertension, reduced sleep duration, and diabetes.^[13]^ Although our knowledge of circadian rhythm-related health problems has expanded, no previous studies have explored the relationships of CircS with NAFLD and liver fibrosis.

To better understand the potential association of CircS with NAFLD and liver fibrosis, we analyzed data from multiple cycles of the National Health and Nutrition Examination Survey (NHANES). This research aims to provide fresh insights into the management and treatment of NAFLD and liver fibrosis.

## 2. Materials and methods

### 2.1. Data source

This study is a cross-sectional analysis, utilizing data from the NHANES database for the period 2017 to 2020.03. The NHANES database provides a representative reflection of the health and nutritional status of the U.S. population, with all participants having provided written informed consent. Since the NHANES database is publicly available, ethical review for this study was waived.

### 2.2. The study cohort

**Figure 1.**
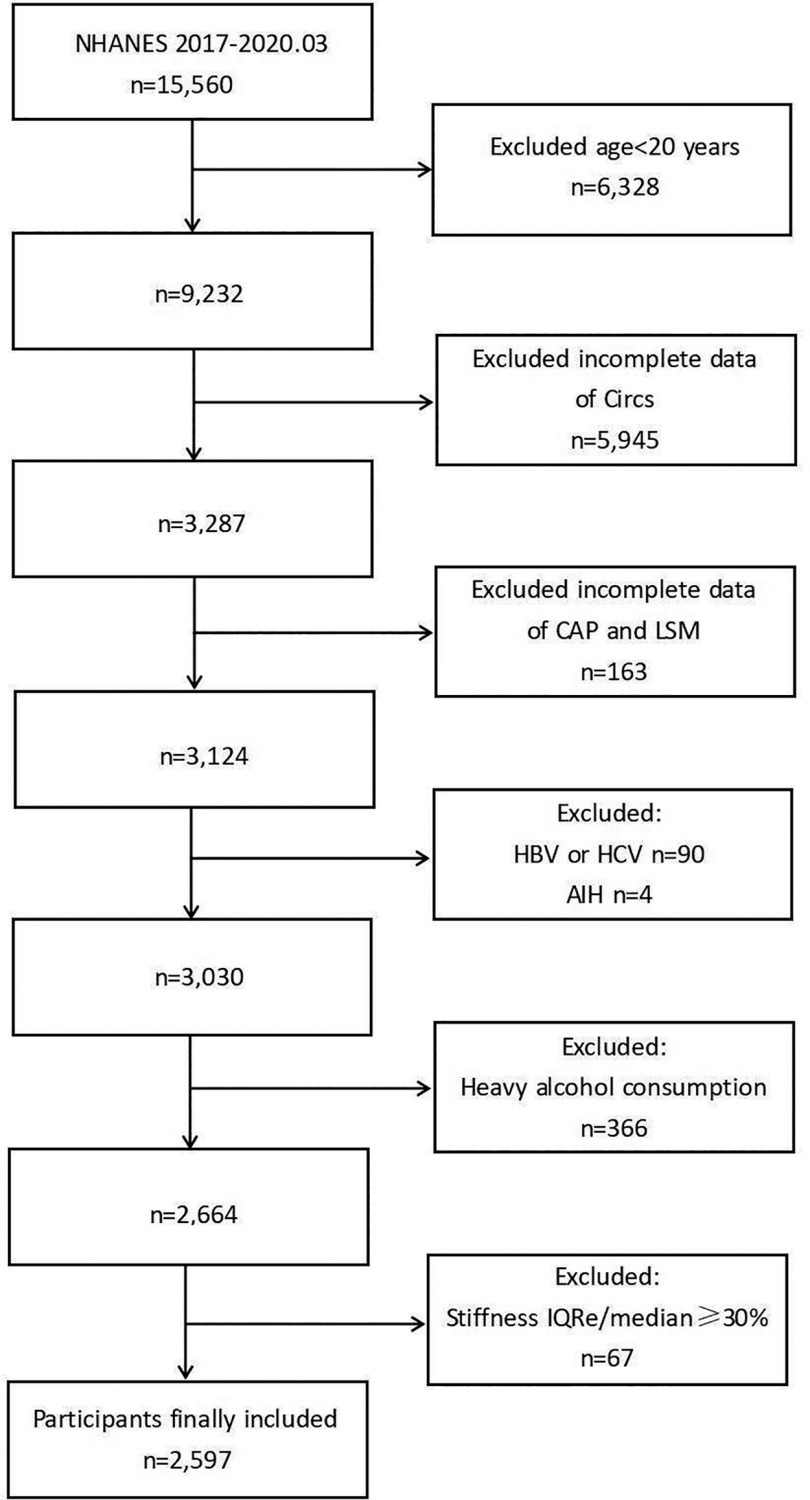
Flowchart of participants selection from NHANES 2017 to 2020.03.

A total of 15,560 participants were surveyed. We excluded those under 20 years of age (n = 6328), those without CircS information (n = 5945), and those who did not undergo LUTE testing (n = 163). We also excluded participants with hepatitis B or C (n = 90), autoimmune hepatitis (n = 4), heavy alcohol use^[14]^ (males > 30 g/day, females > 20 g/day, n = 366), and those with unreliable liver stiffness measurements (LSM interquartile range/median ratio of ≥30%, n = 65). The final sample included 2597 participants, as outlined in Figure 1.

### 2.3. Study variables

#### 2.3.1. NAFLD and liver fibrosis (dependent variables)

Following previous studies,^[15,16]^ a median controlled attenuation parameter value of ≥274 dB/m was utilized as a marker for hepatic steatosis, while a median LSM value of ≥8.0 kPa indicated liver fibrosis (≥F2). Participants included in the analysis were limited to those with reliable LUTE tests, defined as an LSM interquartile range/median ratio of <30%.

#### 2.3.2. CircS (exposure variable)

The diagnostic criteria for CircS required meeting at least 4 of the following: waist circumference ≥88 cm for women or ≥102 cm for men; triglycerides ≥150 mg/dL or lipid-lowering medication use; HDL-C <40 mg/dL for men or <50 mg/dL for women, or lipid-lowering treatment; systolic BP ≥130 mm Hg, diastolic BP ≥85 mm Hg, or antihypertensive medication use; fasting glucose ≥100 mg/dL or diabetes medication use; sleep duration ≤6 h/d; depressive symptoms, indicated by a Patient Health Questionnaire-9 score ≥10.

### 2.4. Covariates

Drawing from previous studies, we accounted for potential confounding variables in our multivariable adjustment models.^[17–19]^ Demographic information included age, gender, race/ethnicity, income-to-poverty ratio (PIR), marital status, and educational level. Clinical data included C-reactive protein (CRP) and serum ferritin. The questionnaire covered smoking status (defined as having smoked at least 100 cigarettes in one’s lifetime) and diabetes status. Dietary information included energy intake, sugar intake, fat intake, and water intake.

### 2.5. Statistical analysis

All statistical analyses were weighted following NHANES analytic guidelines, and multiple imputation was applied to address missing covariate data. Baseline characteristics were presented as mean ± standard deviation for continuous variables and frequency (percentage) for categorical variables. Linear regression was used to compare continuous variables, while categorical variables were compared using Chi-square tests. To evaluate the association of CircS with NAFLD and liver fibrosis, 3 multivariable logistic regression models were implemented: Model 1 was unadjusted, Model 2 accounted for age, sex, race, education, PIR, and marital status, and Model 3 included additional adjustments for CRP, ferritin, smoking status, diabetes, and intake of energy, sugar, fat, and water. Two-sided *P* < .05 was considered to indicate statistical significance. All analyses were performed using R (version 4.2; The R Foundation for Statistical Computing, The R Project, Vienna, Austria) or EmpowerStats (version 5.0; X&Y Solutions, Milford).

## 3. Results

### 3.1. Participant characteristics

In total, 2597 individuals from the NHANES 2017 to 2020.03 cycle were selected for the analysis. The majority of baseline characteristics demonstrated statistically significant variations across groups based on the presence of CircS. As shown in Table 1, the prevalence of NAFLD and liver fibrosis was elevated in patients with CircS, at 71.38% and 16.64%, respectively. Furthermore, these individuals were more likely to be female (56.77%) and tended to have higher educational attainment (76.83%), along with elevated serum ferritin levels (174.80 ± 198.61 ng/mL), and CRP concentrations (5.44 ± 7.80 mg/L). Compared to those non-CircS, individuals with CircS were older (50.36 ± 17.19 years), had lower income levels (2.38 ± 1.53), and were more likely to be smokers (47.12%) and have diabetes (32.19%). Additionally, Tables 2 and 3 illustrate that the prevalence of CircS was notably higher among participants diagnosed with NAFLD and liver fibrosis.

**Table 1:** Characteristics of participants by categories of circadian syndrome: NHANES 2017 to 2020.03.

Characteristics	Overall	Non-circadian syndrome	Circadian syndrome	*P*-value
	n = 2597	n = 1954	n = 643	
Age (yr)	50.36 ± 17.19	48.78 ± 17.56	55.14 ± 15.05	<.001
<60 (%)	64.57	67.35	56.14	
≥60 (%)	35.43	32.65	43.86	
Gender (%)				<.001
Male	48.98	50.87	43.23	
Female	51.02	49.13	56.77	
Race (%)				.016
Mexican American	13.95	12.54	14.62	
Other Hispanic	10.63	10.08	12.29	
Non-Hispanic White	33.73	32.91	36.24	
Non-Hispanic Black	24.87	25.79	22.08	
Other races	17.71	18.68	14.77	
Education (%)				<.001
Less than high school	18.17	16.53	23.17	
High school and above	81.83	83.47	76.83	
PIR	2.62 ± 1.61	2.70 ± 1.63	2.38 ± 1.53	<.001
<1.3 (%)	26.26	24.92	30.33	
1.3–3.5 (%)	41.70	40.58	45.10	
>3.5 (%)	32.04	34.49	24.57	
Marital status (%)				<.001
Married/living with partner	58.88	58.96	58.63	
Widowed/divorced/separated	21.95	20.11	27.53	
Never married	19.18	20.93	13.84	
Smoking (%)				<.001
Yes	48.98	38.08	47.12	
No	51.02	61.92	52.88	
Diabetes (%)				<.001
Yes	82.94	12.08	32.19	
No	17.06	87.92	67.81	
Activity intensity (min)	368.12 ± 659.13	346.85 ± 527.13	432.76 ± 951.81	.014
CRP (mg/L)	4.08 ± 7.52	3.63 ± 7.37	5.44 ± 7.80	<.001
Ferritin (ng/mL)	155.18 ± 169.09	148.72 ± 157.70	174.80 ± 198.61	<.001
Total daily energy intake (kcal)	2032.69 ± 907.65	2044.25 ± 892.94	1997.58 ± 950.80	.074
Total daily sugar intake (g)	103.94 ± 72.51	103.25 ± 70.51	106.06 ± 78.30	.700
Total daily fat intake (g)	84.76 ± 45.96	85.37 ± 45.61	82.93 ± 46.99	.110
Total daily moisture intake (g)	2777.65 ± 1469.32	2757.28 ± 1440.67	2839.55 ± 1552.68	.290
Stratified by CAP (%)				<.001
<274	56.76	66.02	28.62	
≥274	43.24	33.98	71.38	
Stratified by LSM (%)				<.001
<8.0 (%)	90.53	92.89	83.36	
≥8.0 (%)	9.47	7.11	16.64	

Mean ± SD for continuous variables: the *P* value was calculated by the weighted linear regression model; (%) for categorical variables: the *P* value was calculated by the weighted Chi-square test.

CAP = controlled attenuation parameter, CRP = C-reactive protein, LSM = liver stiffness measure, PIR = the ratio of income to poverty.

**Table 2:** Characteristics of participants by categories of nonalcoholic fatty liver disease: NHANES 2017 to 2020.03.

Characteristics	Overall	CAP < 274	CAP ≥ 274	*P*-value
	n = 2597	n = 1474	n = 1123	
Age (yr)	50.36 ± 17.19	48.00 ± 17.75	53.45 ± 15.91	<.001
<60 (%)	64.57	69.20	58.50	
≥60 (%)	35.43	30.80	41.50	
Gender (%)				<.001
Male	48.98	45.93	52.98	
Female	51.02	54.07	47.02	
Race (%)				<.001
Mexican American	13.95	9.70	17.45	
Other Hispanic	10.63	10.92	10.24	
Non-Hispanic White	33.73	31.89	36.15	
Non-Hispanic Black	24.87	28.56	20.04	
Other races	17.71	18.93	16.12	
Education (%)				.103
Less than high school	18.17	17.10	19.59	
High school and above	81.83	82.90	80.41	
PIR	2.62 ± 1.61	2.61 ± 1.62	2.63 ± 1.59	.821
<1.3 (%)	26.26	26.66	25.73	
1.3–3.5 (%)	41.70	40.43	43.37	
>3.5 (%)	32.04	32.90	30.90	
Marital status (%)				<.001
Married/living with partner	58.88	54.21	65.00	
Widowed/divorced/separated	21.95	22.80	20.84	
Never married	19.18	23.00	14.16	
Smoking (%)				.009
Yes	48.98	38.13	43.19	
No	51.02	61.87	56.81	
Diabetes (%)				<.001
Yes	82.94	9.57	26.89	
No	17.06	80.43	73.11	
Activity intensity (min)	368.12 ± 659.13	356.23 ± 646.66	383.73 ± 675.13	.005
CRP (mg/L)	4.08 ± 7.52	3.36 ± 7.07	5.01 ± 7.97	<.001
Ferritin (ng/mL)	155.18 ± 169.09	140.40 ± 152.00	174.57 ± 187.51	<.001
Total daily energy intake (kcal)	2032.69 ± 907.65	1998.89 ± 903.71	2077.05 ± 911.29	.030
Total daily sugar intake (g)	103.94 ± 72.51	102.41 ± 71.40	105.96 ± 73.93	.217
Total daily fat intake (g)	84.76 ± 45.96	82.95 ± 46.03	87.15 ± 45.78	.021
Total daily moisture intake (g)	2777.65 ± 1469.32	2713.36 ± 1511.20	2862.03 ± 1408.68	.011
Circadian syndrome (%)				<.001
Yes	24.76	12.48	40.87	
No	75.24	87.52	59.13	

Mean ± SD for continuous variables: the *P* value was calculated by the weighted linear regression model; (%) for categorical variables: the *P* value was calculated by the weighted Chi-square test.

CAP = controlled attenuation parameter, CRP = C-reactive protein, PIR = the ratio of income to poverty.

**Table 3:** Characteristics of participants by categories of liver fibrosis: NHANES 2017 to 2020.03.

Characteristics	Overall	LSM < 8.0	LSM ≥ 8.0	*P*-value
	n = 2597	n = 2351	n = 246	
Age (yr)	50.36 ± 17.19	49.79 ± 17.24	55.74 ± 15.74	<.001
<60 (%)	64.57	65.80	52.85	
≥60 (%)	35.43	34.20	47.15	
Gender (%)				.038
Male	48.98	48.32	55.28	
Female	51.02	51.68	44.72	
Race (%)				.051
Mexican American	13.95	12.80	15.45	
Other Hispanic	10.63	10.63	10.57	
Non-Hispanic White	33.73	33.09	39.84	
Non-Hispanic Black	24.87	25.22	21.54	
Other races	17.71	18.25	12.60	
Education (%)				.568
Less than high school	18.17	18.03	19.51	
High school and above	81.83	81.97	80.49	
PIR	2.62 ± 1.61	2.62 ± 1.61	2.57 ± 1.60	.710
<1.3 (%)	26.26	26.41	24.80	
1.3–3.5 (%)	41.70	41.17	46.75	
>3.5 (%)	32.04	32.41	28.46	
Marital status (%)				.051
Married/living with partner	58.88	58.53	62.20	
Widowed/divorced/separated	21.95	21.69	24.39	
Never married	19.18	19.78	13.41	
Smoking (%)				.180
Yes	48.98	39.90	44.31	
No	51.02	60.10	55.69	
Diabetes (%)				<.001
Yes	82.94	14.16	44.72	
No	17.06	85.84	55.28	
Activity intensity (min)	368.12 ± 659.13	346.85 ± 527.13	432.76 ± 951.81	.014
CRP (mg/L)	4.08 ± 7.52	3.63 ± 7.37	5.44 ± 7.80	<.001
Ferritin (ng/mL)	155.18 ± 169.09	148.72 ± 157.70	174.80 ± 198.61	<.001
Total daily energy intake (kcal)	2032.69 ± 907.65	2044.25 ± 892.94	1997.58 ± 950.80	.074
Total daily sugar intake (g)	103.94 ± 72.51	103.25 ± 70.51	106.06 ± 78.30	.700
Total daily fat intake (g)	84.76 ± 45.96	85.37 ± 45.61	82.93 ± 46.99	.110
Total daily moisture intake (g)	2777.65 ± 1469.32	2757.28 ± 1440.67	2839.55 ± 1552.68	.290
Circadian syndrome (%)				<.001
Yes	24.76	22.80	43.50	
No	75.24	77.20	56.50	

Mean ± SD for continuous variables: the *P* value was calculated by the weighted linear regression model; (%) for categorical variables: the *P* value was calculated by the weighted Chi-square test.

CRP = C-reactive protein, LSM = liver stiffness measure, PIR = the ratio of income to poverty.

### 3.2. Logistic regression

As shown in Table 4, across all models, CircS was positively associated with NAFLD and liver fibrosis. The odds ratio for NAFLD in the CircS group compared to the non-CircS group was 4.85 (95% CI: 3.99–5.89, *P* < .001), while the odds ratio for liver fibrosis was 1.73 (95% CI: 1.29–2.34, *P* < .001). In addition, except for the nonsignificant association between the ≥6 CircS component group and liver fibrosis in Model 3, all other analyses indicated a positive relationship between CircS components and NAFLD or liver fibrosis.

**Table 4:** Association of circadian syndrome with NAFLD and liver fibrosis.

Variables	Model 1		Model 2		Model 3	
	OR (95% CI)	*P*-value	OR (95% CI)	*P*-value	OR (95% CI)	*P*-value
NAFLD						
Circadian syndrome						
No	1		1		1	
Yes	4.85 (3.99–5.89)	<.001	4.64 (4.28–5.04)	<.001	4.25 (3.44–5.24)	<.001
Components of circadian syndrome						
<4	1		1		1	
4	3.42 (2.73–4.28)	<.001	3.31 (2.63–4.16)	<.001	2.99 (2.36–3.80)	<.001
5	5.01 (3.55–7.08)	<.001	4.92 (3.45–7.03)	<.001	4.21 (2.92–6.07)	<.001
≥6	3.71 (1.72–7.97)	<.001	3.40 (1.55–7.37)	.002	2.70 (1.18–6.16)	.019
Liver fibrosis						
Circadian syndrome						
No	1		1		1	
Yes	2.61 (1.99–3.41)	<.001	2.38 (2.13–2.66)	<.001	1.73 (1.29–2.34)	<.001
Components of circadian syndrome						
<4	1		1		1	
4	1.96 (1.44–2.66)	<.001	1.79 (1.31–2.45)	<.001	1.43 (1.03–1.99)	.035
5	2.38 (1.61–3.51)	<.001	2.17 (1.46–3.23)	<.001	1.60 (1.04–2.44)	.031
≥6	3.01 (1.35–6.71)	.007	2.63 (1.16–5.96)	.021	1.52 (0.62–3.72)	.354

Model 1: adjusts for none.

Model 2: adjusts for age, gender, race, PIR, education, marital status.

Model 3: adjusts for age, gender, race, PIR, education, marital status, smoking, diabetes, activity intensity, CRP, ferritin, energy, sugar, fat, moisture.

CI = confidence interval, CRP = C-reactive protein, NAFLD = nonalcoholic fatty liver disease, OR = odds ratio, PIR = the ratio of income to poverty.

### 3.3. Subgroup and interaction analyses

**Figure 2.**
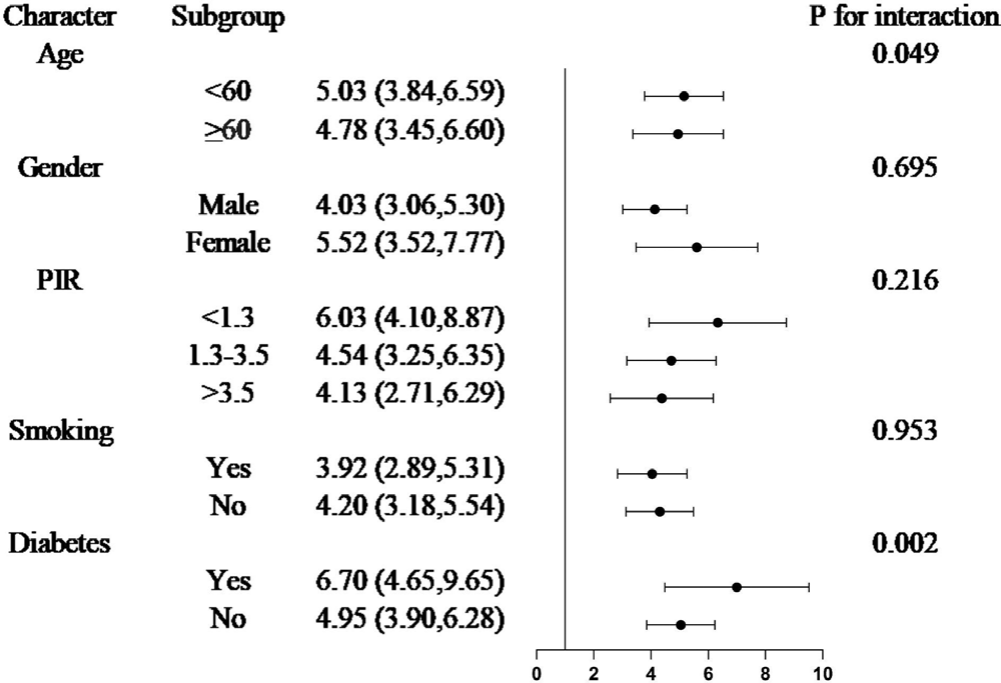
Subgroup and interaction analysis of the association between Circs and NAFLD. CI = confidence interval; OR = odds ratio; Circs = circadian syndrome; NAFLD = nonalcoholic fatty liver disease. Statistical analysis was performed in Model 3.

**Figure 3.**
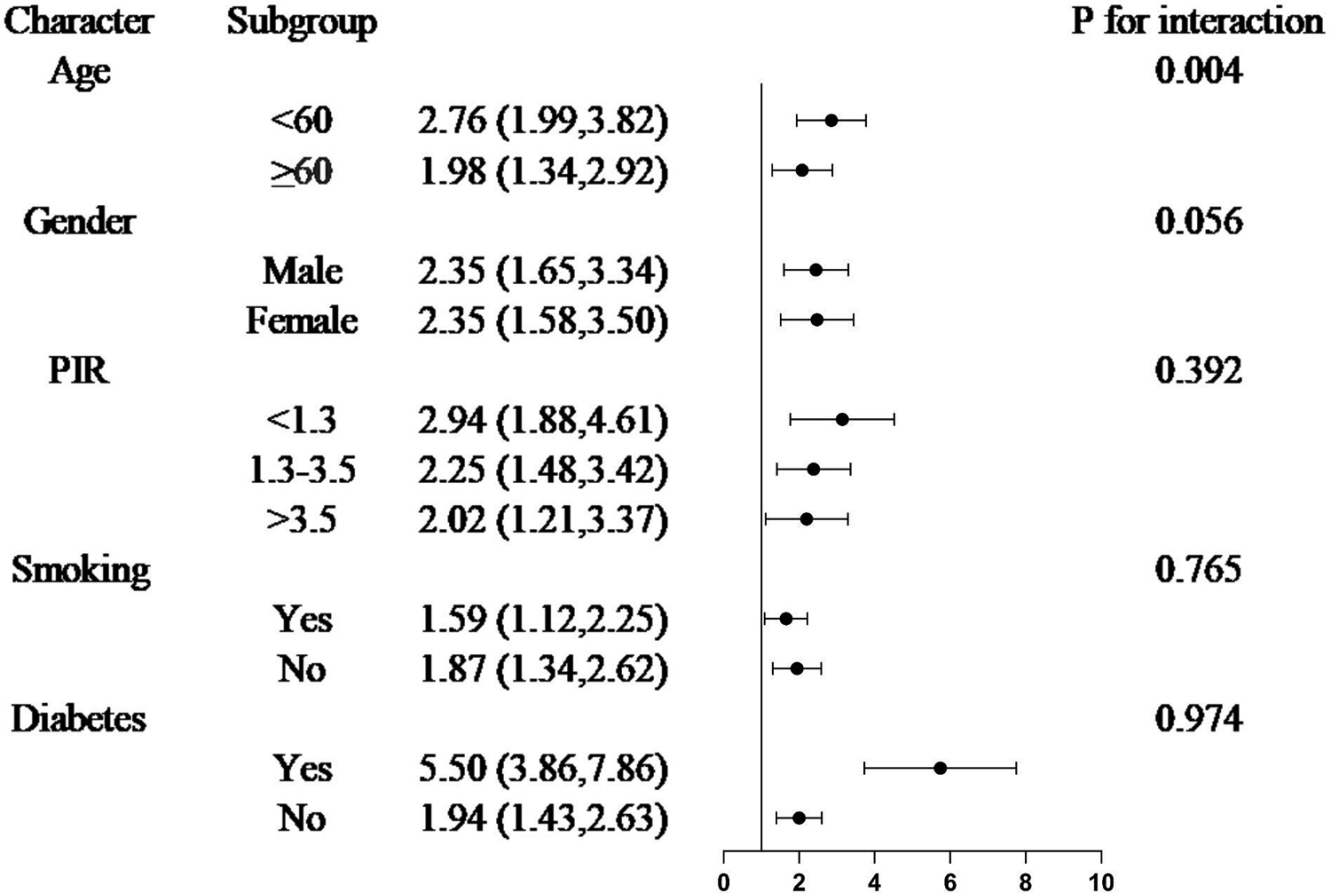
Subgroup and interaction analysis of the association between Circs and liver fibrosis. CI = confidence interval; Circs = circadian syndrome; OR = odds ratio. Statistical analysis was performed in model.

In the subgroup analyses (Figs. 2 and 3), we focused on age, sex, PIR, smoking status, and diabetes. CircS had a stronger positive association with NAFLD and liver fibrosis in younger participants (<60 years, *P* = .049; *P* = .004). Furthermore, a stronger association was observed between CircS and NAFLD among participants with diabetes (*P* = .002). No significant differences were observed in other subgroup analyses.

## 4. Discussion

This cross-sectional study examined the associations of CircS with NAFLD and liver fibrosis, using data from the NHANES database covering 2017 to 2020.03. The findings suggest that CircS is significantly linked to an increased prevalence of NAFLD or liver fibrosis, especially among individuals younger than 60. Furthermore, the presence of diabetes intensified the connection between CircS and NAFLD.

The circadian rhythm system plays a critical role in maintaining metabolic health by regulating gut microbiota, hormones, and inflammation, which are vital for disease prevention and treatment.^[20–23]^ With the growing prevalence of modern lifestyle factors such as light pollution, shift work, and dietary changes, the impact of circadian rhythm disruption on health has become increasingly apparent.^[24–26]^ For example, shift workers with similar body mass index as daytime workers are more likely to develop metabolic disorders.^[27]^ CircS is a clinical syndrome characterized by circadian rhythm disruption and several associated chronic conditions, making it easier to apply in clinical practice. Previous research has found positive correlations between CircS and chronic diarrhea, kidney stones, frailty, and stroke.^[28–31]^

The liver is a crucial metabolic organ, and its functions are closely tied to circadian rhythms. Circadian regulation ensures that liver metabolic activities are synchronized with physiological timing by modulating the expression of key metabolic enzymes and transcription factors. Many major liver functions, particularly those related to energy balance and nutrient metabolism, exhibit rhythmic fluctuations. For example, glucose transporters and glucagon receptors involved in glucose metabolism show rhythmic expression throughout the day.^[32,33]^ Similarly, key enzymes involved in lipid metabolism, such as glycerol-3-phosphate acyltransferase, exhibit circadian rhythmicity.^[34]^ In this study, we observed the association of CircS with NAFLD and liver fibrosis. However, the relationship between sleep duration and NAFLD or liver fibrosis remains controversial. Some studies have found that NAFLD patients experience poor sleep quality and delayed sleep onset,^[35]^ similar to findings in cirrhosis patients.^[36]^ A study by Um YJ et al, which followed 86,530 adults without baseline NAFLD or fibrosis over 3.6 years, found that reduced sleep duration or poor sleep quality increased the risk of developing NAFLD.^[37]^ Conversely, some studies have reported no significant association between sleep duration and the risk of NAFLD.^[38]^ This discrepancy may be because sleep duration, as part of CircS diagnosis, does not fully capture the complexity of circadian rhythm disruption.

The association between age and NAFLD has also been debated. For example, a retrospective study of 1509 NAFLD patients found a “U-shaped” relationship between age and NAFLD prevalence, with a peak in men aged 40 to 49 and women aged 60 to 69.^[39]^ A meta-analysis on global NAFLD prevalence found that the rate increases continuously across all age groups from 30 to 79 years.^[40]^ Moreover, studies suggest that aging may accelerate the progression of liver fibrosis.^[41]^ In our subgroup analysis, we found that CircS was more strongly associated with NAFLD and liver fibrosis in participants younger than 60 years, possibly due to greater social stress, unhealthy dietary habits, and shift work.^[42–44]^ Additionally, we found a stronger correlation between CircS and NAFLD in participants with diabetes, consistent with previous findings. For instance, a cohort study of 18,503 participants reported that approximately 30% of diabetic patients also had NAFLD, highlighting the positive association between the 2.

This study has several notable strengths, such as the use of a large, nationally representative sample and a multistage random sampling approach, which improves the reliability and generalizability of the findings. To our knowledge, CircS, as a newly emerging concept related to metabolism, has not been previously examined in relation to NAFLD and liver fibrosis using a representative sample of U.S. adults. However, a few limitations should be acknowledged. The cross-sectional nature of the study prevents us from establishing causal relationships between CircS and NAFLD or liver fibrosis. Furthermore, the limitations of the NHANES database restricted our ability to include all potential factors that could influence NAFLD and liver fibrosis. Future research is necessary to confirm the causal links between CircS and these liver conditions.

## 5. Conclusion

In U.S. adults, CircS shows significant positive associations with both NAFLD and liver fibrosis. Our findings suggest that individuals under 60 years old and those with diabetes may represent high-risk populations requiring greater attention to liver health. However, as this is a cross-sectional study, these observed associations do not imply causation, and the potential bidirectional relationships between circadian dysfunction and liver diseases warrant further investigation through longitudinal studies.

## Acknowledgments

We would like to thank all participants in this study.

## Author contributions

**Conceptualization:** Guanhua Lv.

**Investigation:** Wenzhao Guo, Sijia Li.

**Writing – original draft:** Wenzhao Guo, Yuhang Gao, Meng Mi.

**Writing – review & editing:** Bingjiu Lu, Xinghai Yue, Wenting Zhao, Ran Zhao.
